# Supplementary material for: Patterns of Intron Gain and Loss in Fungi
Source: PLoS Biol. 2004 Nov 30;2(12):e422. doi: 10.1371/journal.pbio.0020422 (PMC532390; doi:10.1371/journal.pbio.0020422)
Supplement: Table S1 — Also available at http://genes.mit.edu/NielsenEtAl/. (4.3 MB ZIP). [file pbio.0020422.st001.zip › NielsenEtAl/html/1228.html]

AN3824.1.NCU05095.1.MG04425.1.FG01104.1


```
 CLUSTAL W (1.82) Multiple Sequence Alignments - Introns Inserted


Sequence 1: NCU05095.1	517 aa
Sequence 2: MG04425.1	518 aa
Sequence 3: FG01104.1	511 aa
Sequence 4: AN3824.1	516 aa
Alignment Length: 534 aa
Number Identitical Residues: 288 aa
Alignment Score (without introns) 13790


MG04425.1 	MAT-SNSQGPAGDLALEILAALDSKGQPLLTADAFPDAKFVEVKSALDRLGSRSMITYKT
NCU05095.1	MAPPTNTTASSEDLATDILAALD-KSQPLLSSDAFPNATFVDLKAALDRLASREMVKYDT
FG01104.1 	MTT--SSSAPSGDLTSQILQALS-EKTPLLSSEVFPTIAFQDIKASLDRLASRSMITYET
AN3824.1  	---------MAADMTNPVLDALSASEGPILSNEAFPSVPSQTVKAALDRLASRLMVEYET
          	          : *::  :* **.:.  *:*: :.**      :*::****.** *: *.*

MG04425.1 	IERDEYTLEPEAEQIAANGSHEARVFEALRQAVEGLSVAELEEAVGDKSVTKLGQGKAFK
NCU05095.1	IERQEALLEPEAEQIAANGSHEARVFEALRKAVEGLSVKELEEAIGDKNVVKIGQGKAFK
FG01104.1 	IDKEEAILEPEAEQIAEHGSHEARVFEALRNAVEGLSIQDLEKAIGDKTVTKVGQGKAFK
AN3824.1  	FNSEVLVVTKEGEDVAANGSHEAKVYQAVLAAMDGLKIKELPEVVG-KENAQLGQGSAFK
          	:: :   :  *.*::* :*****:*::*:  *::**.: :* :.:* *  .::***.***

MG04425.1 	EKWISKSKDGKKLMAV0AESIKDTTREQLLEIQSTHTHPDAKVLTELKKRKLIKPQKVIS
NCU05095.1	EKWISKTKDGK-FTAV~ADKINDTTQEQIKTVQQTKTHPDPKILADLKKRKLIRTQNIFS
FG01104.1 	EKWIKKSDDGK-LVAT0AKSIEDITRAQLQKIKETRTH-EPKVLTDLRKRKLIKMQKVIS
AN3824.1  	KGWIKK--DKDVLRAA~TDSIVDDTQADVKKIQQGGTVTDPNALKELKKRKLVTAKKVIT
          	: **.*  * . : *. :..* * *: ::  ::.  * .:.: * :*:****:  :::::

MG04425.1 	FEVSKGDKFALEIAKEETDLTADMLASGAWKTATFKPYNFDALGAAQDAGALHPLSKVRS
NCU05095.1	FKIHKGPKFALRIPEEATDLTADMIASGAWKTATFKPYNFKALGATQNGGALHPLNKVRQ
FG01104.1 	FKIEKGPKFALEITKEETDLTADMIASGSWKSANFKPYNFKALGADQNAGALHPLNKVRH
AN3824.1  	FKISKGAKYARELVKEETDLTAEMLQNGSWKTATFKPYNFKAMGAPTPSGAFHPLNKVRQ
          	*:: ** *:* .: :* *****:*: .*:**:*.******.*:**   .**:***.*** 

MG04425.1 	EFRQIFFEMGFEEMPTDKYVESGFWNFDALFVPQQHPARDLQDTFYISDPKAAAPPKAR-
NCU05095.1	ELRNIFFEMGFEEMPTDKFVESGFWNFDALFVPQQHPARDLQDTFYISDPKAADFPRAD-
FG01104.1 	EFRSIFFEMGFEEMPTNKFVETGFWNFDALFVPQQHPARDLQDTFYISDPAVGDAPRS--
AN3824.1  	EFRNIFFEMGFEEMPTNRFVETGFWNFDALFVPQQHPARDLQDTFYISDPLTADPPREDP
          	*:*.************:::**:**************************** ..  *:  .

MG04425.1 	--------------DADDKSNYDEYWQNVRQVHETGKFGSIGYRYPWSPDESLRLVLRTH
NCU05095.1	--------------SPDDTKDYQAYWDNVKAVHQDGKYGSIGYRYPWSGDESLRLVLRTH
FG01104.1 	----------------EEGQNTEEYWENIKQVHQDGKFGSIGYRYPWSAEETKRLVLRTH
AN3824.1  	EYDPHRPKTVLKPAGEEKKLDYKEYWENVRQVHENGKYGSIGYRYPWSPEEALRLVLRTH
          	. ..  ..:  ..:. :.  : . **:*:: **: **:********** :*: *******

MG04425.1 	TTAISTAMLHKLASQKGPDGRPPPARYFSIDRVFRNETVDATHLAEFHQVEGVIADYGLT
NCU05095.1	TTAISANMLHKLAATKGPDGRPLPARYFSIDRVFRNETVDATHLAEFHQVEGVIADYGLT
FG01104.1 	TTAISTAMLYKLAAQKGPDGRPPPARYFSIDRVFRNETVDATHLAEFHQVEGVIADYDLT
AN3824.1  	TTSISTYMLHKLAAN------PRPARYFSIDRVFRNEAVDATHLAEFHQIEGVIADFDLT
          	**:**: **:***:       * **************:***********:******:.**

MG04425.1 	LGGLMEFMDIFFGKMGLTGLQYKPAYNPYTEPSMEIFAYH~QGLGKLVEIGNSGMFRPEM
NCU05095.1	LGGLMEFMEIFFGKMGLHDLQFKPAYNPYTEPSMEIFAYH1KGLGKLVEIGNSGMFRAEM
FG01104.1 	LGGLMEFMEAFFAKMGITNLKFKPAYNPYTEPSMEIFSYH~EGLGKLVEIGNSGMFRPEM
AN3824.1  	LGGLIGFMEVFFAKMGIHKLRFKPAYNPYTEPSMEIFGYH~PGLGKWVEIGNSGMFRPEM
          	****: **: **.***:  *::***************.**  **** **********.**

MG04425.1 	LESMGLP~RDMRVYGWGLSLERPTMIK~YAISNIRELLGHKVDLNFIATNPAVRLDKD
NCU05095.1	LESMGLP2KDMRVFGWGLSLERPTMIK~YGISNIRELLGHKVDLEFVQGSPAVLLGQE
FG01104.1 	LEPMGLP~KDMRVFGWGLSLERPTMIK~YKISNIRELLGHKVDLNFIERNPAVRLEKE
AN3824.1  	LEPMGLP~KDMRVYGWGLSLERPTMIK2YGVSNIRELLGHKVDLNFIETNPAVRLEKD
          	**.**** :****:************* * :*************:*:  .*** * ::
```
